# Feasibility, acceptability, concerns, and challenges of implementing supervised injection services at a specialty HIV hospital in Toronto, Canada: perspectives of people living with HIV

**DOI:** 10.1186/s12889-021-11507-z

**Published:** 2021-07-29

**Authors:** Katherine Rudzinski, Jessica Xavier, Adrian Guta, Soo Chan Carusone, Kenneth King, J. Craig Phillips, Sarah Switzer, Bill O’Leary, Rosalind Baltzer Turje, Scott Harrison, Karen de Prinse, Joanne Simons, Carol Strike

**Affiliations:** 1 Dalla Lana School of Public Health, University of Toronto, 155 College St., Toronto, ON M5T 3M7 Canada; 2 School of Social Work, University of Windsor, 167 Ferry Street, Windsor, ON N9A 0C5 Canada; 3 Casey House, 119 Isabella St, Toronto, ON M4Y 1P2 Canada; 4 Department of Health Research Methodology, Evidence, and Impact, McMaster University, 1280 Main Street West 2C Area, Hamilton, ON L8S 4K Canada; 5 Faculty of Health Sciences, University of Ottawa, 190 Laurier Avenue East, Ottawa, ON K1N 6N5 Canada; 6 Ontario Institute for Studies in Education, University of Toronto, 252 Bloor Street West, Toronto, ON M5S 1V6 Canada; 7 Factor-Inwentash Faculty of Social Work, University of Toronto, 246 Bloor Street W, Toronto, ON M5S 1V4 Canada; 8 Dr. Peter AIDS Foundation, 1110 Comox St, Vancouver, BC V6E 1K5 Canada; 9 Providence Health Care - St. Paul’s Hospital, 1081 Burrard St, Vancouver, BC V6Z 1Y6 Canada; 10 Li Ka Shing Knowledge Institute, St. Michael’s Hospital, 209 Victoria St, Toronto, ON M5B 1T8 Canada

**Keywords:** HIV/AIDS, Drug use, Harm reduction, Supervised injection services, Feasibility studies, Hospital utilization

## Abstract

**Background:**

Substance use significantly impacts health and healthcare of people living with HIV/AIDS (PLHIV), especially their ability to remain in hospital following admission. Supervised injection services (SIS) reduce overdoses and drug-related harms, but are not often provided within hospitals/outpatient programs. Leading us to question, what are PLHIV’s perceptions of hospital-based SIS?

**Methods:**

This mixed-methods study explored feasibility and acceptability of implementing SIS at Casey House, a Toronto-based specialty HIV hospital, from the perspective of its in/outpatient clients. We conducted a survey, examining clients’ (*n* = 92) demand for, and acceptability of, hospital-based SIS. Following this, we hosted two focus groups (*n* = 14) and one-on-one interviews (*n* = 8) with clients which explored benefits/drawbacks of in-hospital SIS, wherein participants experienced guided tours of a demonstration SIS space and/or presentations of evidence about impacts of SIS. Data were analysed using descriptive statistics and thematic analysis.

**Results:**

Among survey participants, 76.1% (*n* = 70) identified as cis-male and over half (*n* = 49;54.4%) had been a hospital client for 2 years or less. Nearly half (48.8%) knew about clients injecting in/near Casey House, while 23.6% witnessed it. Survey participants were more supportive of SIS for inpatients (76.1%) than for outpatients (68.5%); most (74.7%) reported SIS implementation would not impact their level of service use at Casey House, while some predicted coming more often (16.1%) and others less often (9.2%). Most focus group/interview participants, believed SIS would enhance safety by reducing health harms (e.g. overdose), increasing transparency between clients and clinicians about substance use, and helping retain clients in care. Debate arose about who (e.g., in/outpatients vs. non-clients) should have access to hospital-based SIS and how implementation may shift organizational priorities/resources away from services not specific to drug use.

**Conclusions:**

Our data showed widespread support of, and need for, hospital-based SIS among client stakeholders; however, attempts to reduce negative impacts on non-drug using clients need to be considered in the balance of implementation plans. Given the increased risks of morbidity and mortality for PLHIV who inject drugs as well as the problems in retaining them in care in a hospital setting, SIS is a key component of improving care for this marginalized group.

**Supplementary Information:**

The online version contains supplementary material available at 10.1186/s12889-021-11507-z.

## Background

People who use drugs (PWUD), especially those who inject, are at risk of a variety of drug-related harms including infections (e.g., skin, soft-tissue), blood-borne diseases (e.g., human immunodeficiency virus (HIV), hepatitis C, hepatitis B), overdose, and mortality [1–4] PWUD are more likely to be hospitalized, and while in hospital frequently continue using drugs [5–8]. Current abstinence-based policies in the majority of hospitals contribute to mandatory patient evictions and/or high rates of leaving against medical advice, often resulting in disruptions in medical treatment, and increased morbidity and mortality [5, 6, 9–11]. Research shows that various controls used to prevent drug use in healthcare environments, may contribute to increased drug-related harms [5, 6, 8, 11, 12]. To date harm reduction services, including supervised injection services (SIS), have been underutilized in hospitals and healthcare settings. SIS offer a professionally supervised space in which people can use pre-obtained drugs in a hygienic environment, access sterile injection equipment, and have rapid access to emergency overdose responses – thereby reducing risks associated with using alone; rushing injections; sharing equipment and/or increasing dosages – as well as referrals to various health and social services [13, 14]. Inclusion of SIS has increasingly been considered [15] and often recommended as a useful complement to existing in-hospital services for PWUD [5, 9–12, 16–20]. The possibility of SIS to maintain client engagement in HIV care is crucial given the increased risks of harm for people living with HIV/AIDS (PLHIV) who inject drugs [21–23]. Since many patients continue to use drugs while in hospital, concerns about overdose and other risks for patients in hospitals will grow as the opioid crisis continues [4, 24–26].

Decisions about SIS implementation often involve feasibility studies and stakeholder engagement. SIS feasibility studies have typically investigated: 1) cost-effectiveness of SIS in a given context [27–32]; 2) acceptability of SIS among various stakeholders; 3) willingness to use SIS among people who inject drugs; and 4) design preferences among potential SIS clients [12, 33–44]. To date, there have been SIS feasibility studies conducted across multiple countries (e.g., Australia, Belgium, Ireland, Mexico, United Kingdom, United States) as well as several Canadian cities, including: Hamilton, London, Ottawa, Toronto, Thunder Bay, Vancouver, and Victoria [34–36, 42, 43, 45–51]. We acknowledge that since the majority of available research on SIS feasibility is of North American (Canadian) or Australian origin, the issues raised may have particular relevance in these contexts (for a detailed review of this literature please see [52]). Feasibility studies also demonstrate that SIS are generally perceived as acceptable programs to many stakeholder groups including PWUD, business owners, neighborhood residents, healthcare providers, social service employees, government and municipal service employees [35, 36, 40–43, 45, 50, 51, 53–58]. Cost and benefit feasibility studies, which account for medical costs of overdoses, HIV and hepatitis C treatment, infections associated with drug injection, and ambulatory and emergency medical services, have shown that SIS are cost-saving programs [27–32, 42, 59].

Feasibility studies lend themselves to uncovering the concerns community members may have and create opportunities for researchers, frontline workers, and decision-makers, to directly and intentionally address these concerns [60]. Among people who inject drugs, existing feasibility studies demonstrate an overwhelming willingness to use SIS with the most common reasons cited being access to sterile equipment, a private space to use drugs, overdose prevention, and a space that is relatively safe from violence and police surveillance [33–35, 37, 40–42, 46–48, 61–63]. Factors associated with a willingness to use SIS include: experiencing homelessness, injecting in public, daily injection drug use, familiarity with supervised consumption services, and previous experiences of overdose – indicating that this programming pre-dominantly attracts vulnerable populations of people who inject drugs [33, 35, 36, 41, 42, 47–49, 62, 63]. Benefits of SIS for community residents include: a decline in discarded injection equipment in the neighborhood and a reduction in overdoses and HIV/hepatitis C infections and transmission. Research has also shown that SIS increases service access for PWUD. Specifically studies show that SIS increases access to primary physical and mental healthcare and addiction services for PWUD as well as referrals and access to a variety of social services including employment programs, housing services, and peer support programs [64–69]. This is a meaningful aspect of SIS, since PWUD often face barriers when accessing services. However, despite these benefits, community residents continue to have concerns about safety and increasing crime in the area surrounding SIS – with many stakeholders expressing a not in my backyard (NIMBY) mentality.

SIS feasibility studies have been conducted in different settings including, in community health centers, hospitals, and at the municipal and regional level [70, 71]. To date, however, few studies have examined SIS feasibility in hospital settings. A notable exception is Ti et al., (2015), a feasibility study looking at PWUD’s willingness to access in-hospital SIS, where the authors found that over two thirds of participants expressed a willingness to use such services [12]. Likewise, little research has focused specifically on PLHIV, with the exception of work at the Dr. Peter Center, a day health program and 24-h nursing care residence for PLHIV in Vancouver, Canada [72]. Research shows that for PLHIV who use drugs, injection-related infections are a serious health risk, due to their heightened susceptibility [21, 22, 72], and HIV infection has also been associated with an increased risk of overdose mortality [23, 73]. Moreover, given the prevalence of active drug use in hospitals [5–7] and that PWUD are more likely than other patients to be discharged against medical advice [7, 74], there is an urgent need for assessing harm reduction responses, such as SIS, in various healthcare environments in order to reduce risks for clients who use drugs. Indeed Dong et al., (2020) found that a hospital integrated SIS can improve client safety (i.e., reducing overdose and drug-related harms) and increase engagement in addiction treatment [75]. In this article, we report findings from a mixed-methods study exploring perceptions of PLHIV hospital clients regarding the feasibility and acceptability of implementing SIS within Casey House, an HIV hospital in Toronto, Canada. Our study contributes to the growing research on the urgent need for expanding harm reduction initiatives in in/outpatient settings, yet also considers both potential benefits and challenges to implementing these services in a healthcare environment from the perspectives of current clients. Given the current gap in research on SIS in hospital settings, there are only a few hospitals worldwide that provide these services (e.g., Royal Alexandra Hospital (Edmonton) [75], St. Paul’s Hospital (Vancouver) [76], Lariboisière Hospital (Paris); Central Hospital (Strasburg) [77, 78]) our study meaningfully adds to the literature by considering the feasibility of implementing these services from the perspective of hospital clients. Moreover, there is a lack of research on the opportunities and challenges of providing SIS specifically for PLHIV which our research addresses.

## Methods

Our SIS feasibility study used the sequential explanatory mixed methods approach which moved through two consecutive phases of data collection and analysis [79–81]: a short quantitative survey (phase 1), and focus group discussions and one-on-one interviews (phase 2) with key stakeholders, which included in/outpatient clients, clinical and non-clinical staff, physicians, volunteers, managers, and board members at Casey House. In this article, we focus on the results of client data only; a separate paper on staff and non-client stakeholder perspectives is currently under development. This study and related protocols were approved by the University of Toronto HIV Research Ethics Board.

### Setting

Casey House, initially established as an AIDS hospice in 1988, is a sub-acute care specialty hospital that addresses the changing care needs of PLHIV. Clients are medically complex and socially vulnerable, including those living with acute mental health illnesses, substance use disorder, cognitive impairment, poverty and unstable housing [82, 83]. With an estimated 80% of clients actively using drugs, the hospital officially adopted a harm reduction policy (2008) and since 2014 has been the primary distributor of harm reduction kits to clients and PWUD in the surrounding community [84, 85]. The need for harm reduction services in this neighbourhood is manifest, as it has the highest number of emergency medical service overdose calls in the city [86]. In 2017 Casey House moved into a new facility that includes a 14-bed inpatient program, which sees approximately 100 admissions per year for sub-acute care for opportunistic infections, stabilization, respite, and palliative care, and is staffed by a multidisciplinary team (e.g., physicians, nurses, social workers, etc.). A new outpatient day health program (DHP) that currently assists approximately 250 clients living with HIV at risk for, or experiencing, deteriorating health, also operates 5 days a week. The DHP provides a combination of one-on-one and group programs and services (e.g., lunch program, clinical groups, social/recreational programming). Clients frequently move between the DHP and inpatient program in response to episodes in their health and care needs.

### Participants and procedures

**Fig. 1:**
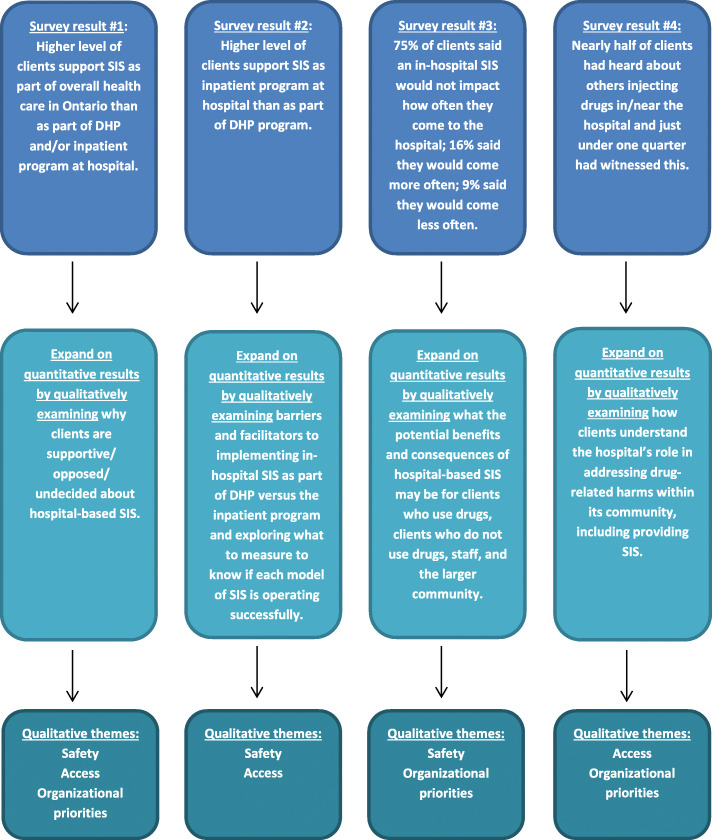
How quantitative survey results relate to the qualitative inquiry and findings

Our study started with a quantitative survey which assessed the demand for and acceptability of SIS. Data analysis of the survey led us to separate focus groups for the next phase by stakeholder type and to build on from the quantitative results by asking specific questions regarding the barriers and facilitators to introducing these services at the hospital, as part of the DHP and/or the inpatient program (e.g., what to measure to know if SIS is operating successfully, potential impact of SIS on clients who use drugs, clients who do not use drugs, staff, the larger community) (See Fig. 1). The survey was followed by qualitative focus groups and interviews that focused on the benefits and concerns around providing a SIS at Casey House and the best ways to introduce such a program at the hospital. We used a follow-up explanations model, which used qualitative data to explain or expand on quantitative results [79, 80]. While the survey provided us with a general understanding of the acceptability and demand for SIS at the hospital, the qualitative data and their analysis allowed us to refine and explain our statistical results by exploring participants’ views in more depth [81].

A short anonymous survey (lasting approximately 10–15 min) that solicited opinions about supervised injection services was conducted with 92 clients. Print and digital advertisements announcing the survey were posted at the hospital. At the time of recruitment, approximately 60 clients attended lunch, programs and/or appointments in the building each day and an additional 14 resided on the inpatient floor. Clients were recruited as they entered the front lobby by a research staff member. People who wished to participate were screened for eligibility (i.e., being a current client) and asked for verbal consent. Using Bowen et al.’s (2009) approach to feasibility studies as those studies that are “conducted to measure one or more of the following elements: acceptability, service use, demand, implementation, practicality, adaptation, integration, expansion, and/or limited efficacy testing” [60], our focus in the survey was to examine acceptability and demand factors. Acceptability is defined as the attitudes about SIS and likely response to it by intended clients as well as those who might be potentially involved in implementing the program [87]. The survey, programmed using Qualtrics online software, included questions about socio-demographics, lifetime and current injection drug use and risk experiences, client’s self-reported knowledge of, and attitudes towards, SIS, perceived demand for and utilization of SIS, and opinions about the acceptability of providing on-site SIS, both as part of the DHP and for inpatients. The survey was conducted using computer-assisted personal interviewing and questions were adapted from previous research [42, 88] (see Supplementary file 1). Data were collected between 12/2018 and 03/2019. No identifying information was collected on the survey – allowing participants to remain anonymous – and $10 compensation was offered for time and effort.

After completing the survey, each participant was asked if they wished to be considered for a future focus group discussion, where facilitators and barriers to the implementation and operation of hospital-based SIS would be explored further. Sixty-one people expressed interest and provided contact information, which was not connected to their previously completed anonymous survey. All were asked to answer a question about whether they were supportive or opposed/undecided about SIS implementation at Casey House. We divided the list of interested people into these two groups and then randomly selected participants for each group. Based on earlier work at Casey House [84, 89], we divided the focus groups by opinion to ensure opportunity for all participants to voice their opinions without creating intergroup conflict.

Each of the two focus groups lasted approximately 2.5 h and included informed consent, a guided tour of a physical mock up SIS space at Casey House, a presentation of research evidence about the impact of SIS, a presentation about the inner-workings of SIS by a local harm reduction worker, a question and answer session and a 15-min break before a semi-structured focus group discussion (lasting approximately 1 h) (for detailed description of methods see [90]). Some members of the research team had lived experience; however for privacy reasons we do not identify who they are. At least one team member with lived experience was involved in all aspects of data collection including conducting the focus groups and interviews. In addition, we had individuals with lived experience of working at and using SIS lead the Q&A portion of the focus groups. The focus group guide developed for this study, included questions about the following topics: impressions of the physical mock up SIS space, opinions regarding the potential implementation of SIS at Casey House, how SIS programming may impact various clients (i.e., clients who currently use drugs, those who do not use, and those who have a history of drug use) and/or how clients use Casey House, as well as the benefits and/or drawbacks of introducing SIS programming (see Supplementary file 2). Participants were provided with $30 honorarium and two transit vouchers, for their time and effort. Of those recruited, five participants who were undecided or opposed to SIS attended the first focus group (FG1) and nine participants in favour of SIS attended the second session (FG2).

Finally, we conducted one-on-one semi-structured interviews with current or recent inpatients (*n* = 8) regarding their views on a potential SIS. Interviews were used to ensure that inpatients too ill to attend a focus group could also express their opinions. Recruitment started with the pool of interested participants from above, who fit eligibility criteria of a current or recent (within the past 6 months) inpatient stay, and was supplemented with the help of hospital staff. Interviews lasted approximately 1.5 h, were completed in a private room at the hospital, and followed an abbreviated focus group format, which included: consent, a short overview of how SIS typically work, photographs of the physical mock up SIS space, evidence regarding benefits and consequences of SIS, and a question and answer session. The subsequent interview focused on topics similar to those addressed during focus groups, but concentrated on SIS as part of the inpatient program, including bedside services (see Supplementary file 3). All participants of focus groups and interviews were asked to complete a brief socio-demographic questionnaire.

### Data analysis

Survey responses and socio-demographic data were imported into SPSS version 26 and analyzed using basic descriptive statistics (e.g., means, standard deviations). Qualitative focus group and interview data were digitally recorded, professionally transcribed, verified for completeness, corrected and uploaded into NVivo 12 qualitative data management software. Members of the research team read (and reread) interview transcripts, to iteratively and inductively develop the coding scheme. Data were analyzed using a thematic analysis approach [91], whereby resultant codes were sorted and collated into major themes, which were refined in the process of team discussion and by periodically returning to raw data to ensure that themes were consistent with participants’ experiences/narratives. Quantitative and qualitative findings were actively compared and contrasted across various themes, with a focus on how qualitative findings added nuance to what was observed on a larger scale in the quantitative survey. A small part of our research team, consisting of academic professionals with years of experience conducting harm reduction research and those with lived experienced, worked on coding and data analysis. Preliminary coding and analysis were presented to the larger research team, which included members with lived experience, to gain a wider perspective on the findings. In this paper, survey results are clarified and expanded upon using quotes with the following identification scheme, focus group (FG)# or interview (INT)#.

## Results

### Survey results

Among survey participants (*n* = 92), 79 (85.9%) were DHP clients and 13 (14.1%) were inpatients. Seventy-six percent of clients (*n* = 70) who completed surveys identified as cis male and just over half (*n* = 49, 54.4%) had been a Casey House client for 2 years or less (Table 1). When asked about drug use, 40.2% (*n* = 37) of participants reported ever injecting drugs during their lifetime; of these 40.5% (*n* = 15) stated that they had injected drugs in the 6 months prior to the survey; and of the latter group 73.3% (*n* = 11) were current injectors, defined as having injected in the past 30 days (groups are not mutually exclusive) (Table 2).

**Table 1:** Client survey demographic characteristics

Characteristic (*n* = 92 unless otherwise stated)	N	%
Current program of client		
Day Health Program	79	85.9
Inpatient	13	14.1
Gender		
Cis male	70	76.1
Cis female	15	16.3
Other (transgender/non-binary/gender fluid)	7	7.6
Length of time as Casey House client (*n* = 90)		
2 years or less	49	54.4
3 to 10 years	22	24.4
11 to 19 years	6	6.7
20 years or more	13	14.4

**Table 2:** Client survey descriptive characteristics of substance use

Characteristic	N	%
Lifetime injection (*n* = 92)		
Yes	37	40.2
No	55	59.8
Injected drugs in the past 6 months (*n* = 37)		
Yes	15	40.5
No	22	59.5
Injected drugs in the past 30 days (*n* = 15)		
Yes	11	73.3
No	4	26.7
Types of drugs injected (past 30 days) (*n* = 11)		
Stimulants only	2	18.1
Opioids only	5	45.5
Both stimulants and opioids	4	36.4
Places where injected (past 6 months) (*n* = 15)		
Public space	7	46.7
SIS	4	26.7
Casey House	6	40.0
Injection-related experiences (past 6 months) (*n* = 15)		
Witnessed an overdose	8	53.3
Abscesses and/or infections	6	40.0
Rushed an injection	6	40.0
Had naloxone when needed	6	40.0
Collapsed vein	4	26.7
Harassed by police	4	26.7
Robbed/attacked while injecting	4	26.7
Experienced an overdose	3	20.0
Used needle used by someone else	3	20.0
Used other equipment used by someone else	3	20.0
Reasons to inject (*n* = 15)		
For pleasure	14	93.3
To help manage emotional/psychological problems	11	73.3
To help manage physical pain	6	40.0
Ever heard of other clients inject in/near Casey House (*n* = 86)		
Yes	42	48.8
No	44	51.2
Ever seen other clients inject in/near Casey House (*n* = 89)		
Yes	21	23.6
No	68	76.4

When asked how knowledgeable they were about SIS, just under half of the survey participants (*n* = 44, 48.9%) self-reported that they had some to average level of knowledge, and another 31.1% (*n* = 28) said they were fairly to very knowledgeable prior to participating in the study (Table 3). Survey results and focus group/interview data showed that there was support for SIS in general and specifically at Casey House, but with a minority of clients expressing outright opposition to the idea. When asked if SIS should be made available to PWUD as part of state funded healthcare services, 88% (*n* = 81) of survey participants were supportive (see Table 3). Survey data showed that participants were more supportive of SIS for inpatient clients (*n* = 70; 76.1%) than they were of SIS for DHP clients (*n* = 63; 68.5%). Moreover 17.4% (*n* = 16) and 14.1% (*n* = 13) of survey participants were undecided about the acceptability of providing SIS in the DHP and the inpatient program, respectively. A relatively small group of survey participants were opposed to SIS implementation in the DHP (*n* = 13; 14.1%) and even fewer were opposed for the inpatient program (*n* = 9; 9.8%). No significant difference was found, using Pearson’s chi-square tests (chi-square = 3.307; DF = 2; *p* = .191), when comparing levels of support for SIS and lifetime injection drug use.

**Table 3:** Client survey knowledge and acceptability of SIS

Characteristic (*n* = 92 unless otherwise stated)	N	%
Knowledge of SIS (*n* = 90)		
None to very little knowledge	18	20.0
Some to average knowledge	44	48.9
Fairly to very knowledgeable	28	31.1
SIS should be made available as part of overall healthcare in Ontario		
Agree	81	88.0
Disagree	6	6.5
Undecided	5	5.4
SIS should be made available as part of the Inpatient Program		
Agree	70	76.1
Disagree	9	9.8
Undecided	13	14.1
SIS should be made available as part of the Day Health Program		
Agree	63	68.5
Disagree	13	14.1
Undecided	16	17.4
Willingness to use potential SIS at Casey House (*n* = 15)		
Yes	7	46.7
No	4	26.7
Undecided	4	26.7
Reasons for willingness to use SIS at Casey House (*n* = 11)^a^		
Trust staff/hospital	11	100.0
Access to clean sterile injection equipment	11	100.0
Overdose can be prevented or treated	10	90.9
Access referrals for other services	10	90.0
Opportunity to inject indoors	9	81.8
Protection from police	9	81.8
Protection from crime	8	72.7
Reasons for unwillingness to use SIS at Casey House (*n* = 8)^b^		
Already have a preferred place to inject	5	62.5
Do not want people to know they use drugs	3	37.5
Can get clean sterile equipment already	3	37.5
Currently do not inject drugs	2	25.0
Too far to travel	1	12.5
Worried their use of SIS will not be kept confidential/will become part of health record	1	12.5
Clients’ perceptions on how existence of SIS would impact how often they would come to Casey House (*n* = 87)		
Would come more often	14	16.1
Would come less often	8	9.2
No impact	65	74.7

^a^Includes both those who said they would be willing to use SIS and those who are undecided

^b^Includes both those who said they would not be willing to use SIS and those who are undecided

### Focus group and interview results

Sociodemographic data from focus group and interview participants show that most were DHP clients (*n* = 17; 81%), identified as cis male (*n* = 21; 95.5%), almost two-thirds had ever injected drugs (*n* = 18; 61.1%), with one-third of this group having injected in the past 30 days (*n* = 4; 36.4%) (Table 4). Levels of acceptability of providing on-site SIS for DHP (*n* = 13; 59.1%) and inpatient (*n* = 16; 72.7%) clients were similar to those found in survey data, with the former being slightly lower. Focus group and interview data added nuance and dimension to survey opinions, connecting levels of acceptability to perceptions of safety, access, and organizational priorities.

**Table 4:** Socio-demographics, drug use characteristics and acceptability of SIS for focus group and interview participants

(*n* = 22 unless otherwise stated)	N	%
Current program of client		
Day Health Program	17	81.0
Inpatient	4	19.0
Gender		
Cis male	21	95.5
Cis female	1	4.5
Drug use		
Ever used drugs		
Yes	18	81.8
No	4	18.2
Ever injected drugs (*n* = 18)		
Yes	11	61.1
No	7	38.9
Injected drugs in past 30 days (*n* = 11)		
Yes	4	36.4
No	7	63.6
Willing to use SIS at Casey House (*n* = 4)		
Yes	2	50.0
No	2	50.0
SIS should be made available as part of the Inpatient Program		
Agree	16	72.7
Disagree	4	18.2
Undecided	2	9.1
SIS should be made available as part of the Day Health Program		
Agree	13	59.1
Disagree	6	27.3
Undecided	3	13.6

### How will safety be affected by potential hospital-based SIS?

During focus groups and interviews, participants spoke about how SIS implementation at Casey House could affect the safety of clients who use drugs, clients who do not use drugs, and the surrounding community. Perceived direct effects on safety for clients who use drugs included reduced overdoses associated with using drugs alone and reduced transmission of blood borne viruses:

If someone is going in to the bathroom to use … you’d be quite alone and you could be there for a while before anyone knew you were in trouble (INT3)

Being in a hospital is stressing, that you do probably want to go use, and if you could do it supervised and not get … off the street … a dirty needle (INT5)

For inpatients who use drugs, SIS was thought to have the potential to reduce the number of patients who leave hospital before their treatment plan is completed:

I think for inpatient, they won’t be rushing just to get out of the hospital. I think that there’s some people that do rush out … but being like, ‘We still want to take care of you here. We still want you to go back home when you’re healthy and ready and able bodied too. So if you need to use, use here.’ … they won’t feel unwelcome (INT5).

Implementation of hospital-based SIS was also perceived to indirectly increase the safety of clients who use drugs. In particular, participants suggested that SIS might reduce the stigma associated with drug use that encourages people to hide their use: “Interviewer: So if they had a space, they wouldn’t have to – what do you mean by sneak around? Participant: Go sneak around, like, go to the washroom and do it” (INT7). The visibility of SIS was thought to also have the potential to promote safety by allowing open discussion and transparency about drug use between clients and care providers and improve care for people who use drugs:

‘Cause even when I was using it right I wouldn’t tell the doctors I was using. You know what I mean? (INT2)

Getting a relationship where you’re both [patient and physician] honest with each other, because that’s how you’re going to get a great health plan (INT5).

More broadly it was suggested that SIS might be a benefit as it could address a potential gap in programming by moving Casey House beyond simply distributing harm reduction equipment: “‘cause it’s improper to hand somebody a needle, and say ‘get out of the building; inject somewhere else.’” (FG2)*.* Others suggested that open discussion of drug use brought about by the implementation of SIS would also increase safety for inpatients: “[inpatient] staff would certainly know, more likely, about … how much they’re using and be able to take that information and sort of consider other drugs they’re administering the person” (INT3). Finally, SIS was believed to have the potential to improve outcomes following inpatient discharge:

Looking at the discharges, because we do have discharge meetings … So you’re going to go home, and you’re going to use drugs as well, but at what times are you going to, is it going to interact with your prescription drugs or things like that (INT5).

Participants also suggested that on-site SIS might increase safety for clients who are trying to remain abstinent because it could reduce their exposure to drug use by containing drug injection to one part of the building: “if they’re not using drugs, it’s away from them and they’re getting a safe injection and they’re getting what they need, and not affecting the other people” (FG2). Conversely, some participants thought that SIS services would result in more visible drug use and/or intoxication possibly triggering clients who were attempting to abstain from drug use, particularly for inpatients unable to distance themselves:

If I happen to be an inpatient client, and I am not a substance user, I’m coming here … for my own wellbeing. Do I need another issue, with people who are using substances … It’s only thirteen rooms, fourteen rooms … upstairs (FG1).

There was also concern about courtesy stigma, wherein clients, primarily those that did not currently use drugs, expressed uneasiness with the idea of all PLHIV accessing the hospital being associated with SIS and, more broadly, “drug users” – a population that experiences high levels of stigma. Among some clients, there appeared to be discomfort and/or fear that Casey House would be increasingly considered a provider of SIS rather than as a health care facility for PLHIV:

And then we are additionally linked, people who [have] HIV, with the idea that ‘Oh, he has HIV; he is doing drugs as well as he has HIV. You know, that he is gay, blah, blah, blah (FG2)

Don’t make stigma again. This building [is] about HIV … [For a] long time people fight [HIV] stigma. And then [fight] more about injection [drug use], more stigma, more stereotypes, for this building (FG1).

Some participants did not believe that Casey House was the appropriate location for SIS because of concerns about changes to public/neighbourhood safety:

Most of them [general population] would be concerned about, you know, is this going to draw more drug users to my area, is my property safe, you know, is my children going to school safe because of this. Like, there’s concerns for the population around here (INT3).

Participants spoke about the potential for SIS to attract drug dealers*,* create increased exposure to disruptive behaviour or sexual harassment:

Interviewer: Do you think that they would need any sort of added security if they have this kind of service at Casey House?

Participant: I don’t know. There’s always a need … ‘cause there’s going to be more drugs. There’s going to be trafficking (INT8)

When they do inject … they’re not fully in a state of mind … Like, their mind goes wandering … a person could have a bad batch and you know, freak out (INT7)

Sexual inappropriateness, but you know, you might think ‘Oh, you’re touching me. I don’t like this.’ … It may not be much, but it’s wrong (FG2).

These participants stressed that if SIS were implemented increased security would be needed at the hospital: “they need to have security, within the lobby, to make sure that the non-users feel safe and comfortable … That they aren’t going to get assaulted, and they’re not going to be accosted” (FG2). Others dismissed these concerns believing that experience within the hospital has shown that PWUD and non-PWUD clients can occupy the same space without problems:

But right now, at the lunch, there’s some people that use substances, people that don’t use substances, and we all get along … Yeah … So, right now, I don’t want to see challenges where there are no challenges. People come in; they sit together … Just because you’re not a substance user, does not mean you can’t be friends with somebody who is a substance user. But right now, it’s going very well (FG2).

### Who should have access to potential hospital-based SIS?

Focus group and interview participants debated about who the SIS might be designed to serve – inpatients, DHP clients, and/or people who inject drugs in the surrounding community. Some only supported SIS for inpatients since, “If they’re here, to ask for help, to live in the hospital upstairs, and they have this problem [addiction], you have to help them. It’s part of the health program” (FG1), and not for DHP clients because “they can go somewhere else” (FG1). One participant suggested that SIS should only be open to clients because there were concerns about engaging with non-PLHIV:

I don’t want to mix with people who are not HIV positive, in any way. I don’t want to hang out with them. I don’t have sex with them. I don’t hit on them. You know, I … just don’t want to like, risking that somebody will get HIV from me. Ever. (FG2).

A desire for the hospital to retain its focus on PLHIV prompted concern for those opposed to SIS being open to people who inject drugs in the surrounding community and who are not HIV positive, “if we opened it to non-HIV people, there’d be too many people at the injection site” (FG2). Some participants had concerns about loss of confidentiality and discrimination if SIS was open to people who were not Casey House clients and not living with HIV:

I will not feel safe if I know people hanging around, going to know, or come up to me on the street, and shout ‘You have HIV positive.’ right, or something like that. Because it’s happened. It’s happened on our street … (FG2).

Nested in this discussion was an understanding that the demand for SIS in the surrounding area was high and might overwhelm the hospital:

Because, I live in this community. I see the needles around, especially down by Shoppers [drug mart], because I live there. And that’s the only reason why I have a sharps container in my house. Because, I’m out with the dogs; I’m out with nine-year-old kids (FG2).

However, some participants believed that SIS designed only for clients would neglect others in the community who could benefit, thereby reducing the opportunity for the hospital to expand its mandate and contribute to the prevention of HIV infection:

And it is wrong for, to wait for somebody to first get infected, before we look after them. If we can prevent them from getting infected with HIV, we should do our damnedest to prevent it (FG2).

This need for a broader mandate was taken up in another way as some felt that many PLHIV would benefit from SIS, yet clients who use drugs need more harm reduction programming at Casey House.

### Conflicting priorities and where do potential SIS fit at Casey house?

There were conflicting opinions held by focus group and interview participants about whether prioritizing potential hospital-based SIS would pull focus and funding away from PLHIV who do not use drugs. Some believed that failing to prioritize SIS at Casey House would amount to not addressing the evident needs of clients who use drugs. Other participants did not think that SIS fit within Casey Houses’ mission and mandate which was, from its inauguration, to meet the often-complex needs of PLHIV: “don’t do something else, and you think that you can benefit the whole society. No one can do that. Each hospital, they have their own focus” (FG1). A smaller number were morally opposed to a harm reduction approach in general believing that SIS would amount to the hospital supporting illegal substance use that is harmful to the well-being of PLHIV:

That’s all it boils down to, is why is Casey House promoting use of an illegal drug when this is a hospital?.. .People come here to get healthy. They don’t come here to [get] stoned (FG1).

Focus group participants who were opposed to hospital-based SIS expressed concern that these services could shift the organization’s attention and resources away from all PLHIV to a sub-group of those who use drugs: “if you direct some resource to this [SIS], you will have less resources for HIV patients” (FG1). There was worry that already limited resources would be taken away from clients who do not use drugs, when these funds should be focused on HIV-related issues: “can you spend somewhere more relevant to … HIV patients” (FG1). For instance, one participant warned that within this context staff might become so overburdened with running SIS, that “they won’t have the energy to do anything with us, when we need them” (FG1). For some participants, there was a strong sense that they would have nowhere else to go if the hospital environment were to change in a way that they perceived as unfavorable, whereas they believed that there were other places in the city that offered SIS:

Safe injection sites, there are so many, so many around the city. Why have an add-on to Casey House? … Why can’t Casey House focus on … HIV, and make us proud (FG1).

A small number of focus group participants claimed that SIS implementation would lead some to stop coming to, or attend Casey House less often

“So for me, if they want to have SIS, I will come here less, because it’s the influence. I don’t want to see more chaotic individuals here … I would probably stop coming here” (FG1).

Survey data corroborated that a small number of clients claimed that they would attend less often (*n* = 8; 9.2%) whereas 74.7% (*n* = 65) of participants reported that SIS implementation would have no impact on their level of service use, and 16.1% (*n* = 14) would come to Casey House more often if there was SIS.

Opposition to SIS was also linked to a perception that Casey House had more pressing priorities to improve care for PLHIV, including more services for mental health problems and reinstatement of homecare services:

A lot of HIV patients … they have mental issues. Why can’t you focus on that? (FG1)

I have to come here now … I’ve been sick … no one can come and see me because they do not have that program anymore … I couldn’t walk. They still want me to come here

(FG1).

Some feared SIS might have negative impact on the fundraising that is so important to hospital operations:

I think that most of the people who donate to Casey House are … very affluent and they’re not necessarily as open minded to drug users and drug use … I think most of them, are connected to some person in their lives that have died from HIV … So there’s a large sort of gay men contingent to … the people who donate and I don’t know if they all buy into the SIS thing. I think you might potentially lose some fundraising (INT3).

However, others believed that with the appropriate ‘spin’ – including a combination of information and effective communication, donors would not be lost.

In spite of some opinions against SIS at Casey House, survey data showed that the need for these services was substantial. Nearly half of participants (*n* = 42; 48.8%) had ever heard about clients injecting in or near the hospital, and almost one quarter (*n* = 21; 23.6%) had witnessed this. Participants also spoke about their own use onsite, “I [did a slam] and I climbed out the window and ran off?” (FG2). Moreover, survey data showed that there was willingness to use hospital-based SIS. Just under half (*n* = 7; 46.7%) of clients who reported injecting drugs in the past 6 months would consider using SIS at Casey House, while just over one quarter were either undecided (*n* = 4; 26.7%) about or unwilling (*n* = 4; 26.7%) to use onsite SIS. For those who reported potential willingness to use onsite SIS (*n* = 11; includes agreed and undecided), all cited trust in Casey House staff and access to injection equipment as reasons, and then referenced the following other motivations: protection from overdoses (*n* = 10; 90.9%), access to service referral pathways (*n* = 10; 90.9%), opportunity to inject indoors (*n* = 9; 81.8%), protection from police (*n* = 9; 81.8%) and crime (*n* = 8; 72.7%). Among those who stated that they would not use SIS at Casey House (*n* = 8; includes disagreed and undecided), the most common reason provided was that they already had a preferred place to use (*n* = 5; 62.5%) (see Table 3). Comments during interviews and focus groups reflected these various levels of willingness to use potential onsite-SIS:

Interviewer: Do you think inpatients would use a separate space … ?

Participant: Oh absolutely I think they would.

Interviewer: What makes you say that?

Participant: Because I know I would.

(INT6)

Participant: Like yeah, a few friends of mine, they can’t be … near, around nobody.

Interviewer: Okay. So they need to always inject alone. It’s just how they do things.

Participant: Yeah.

(INT8)

So, getting the use of it, getting people to trust it, in that way, especially because you do have the title of hospital. There’s some people that have an image of Casey House being a barrier to that, and so they do probably think, ‘Oh, I’m going to get in, do an assessment and just be asked if I want to go into recovery.’ And it’ll be, I think that there will be some hesitancy from some users (INT5).

Finally, there was support for broader harm reduction programming across participants and most agreed that it did not make sense to provide SIS in isolation. Many participants suggested a wider range of services such as additional harm reduction supplies, peer and outreach programming, harm reduction workers, treatment services, and a harm reduction room (which could encompass any or all of these resources) which was seen as a more promising strategy for sharing harm reduction knowledge: “the harm reduction room, absolutely should have other services. And talk about, creating some partnership” (FG2). In these discussions about a harm reduction room, clients mentioned the need for assistance with the broader social determinants of health such as food security and nutrition, housing, mental health, and creating partnerships with external organizations to accomplish this:

Cascade of care, meaning like, you know, get you into treatment. Get your adherence. If you need other, any social determinants of needs, substance use, home … if you need a home, or social insurance, they [Casey House] can arrange it for you (FG1).

## Discussion

Our research showed that there was widespread support for SIS in general, and specifically at Casey House, with only a minority of clients expressing outright opposition. In addition, these findings shed light on the potential barriers and facilitators of implementing SIS within a HIV hospital. Specifically, client opinions about SIS revolved around how safety, access, and organizational priorities could be affected by the potential addition of these services in their healthcare environment. Our study builds on previous literature in several ways, namely by focusing on SIS feasibility in an under-researched setting, an in/outpatient hospital providing HIV/AIDS care, by engaging a unique population of PLHIV, with both PWUD and non-PWUD clients, and by employing a mixed-methods research design.

Our findings show that many participants see significant safety benefits of SIS for clients who use drugs, including lower risk of overdose (from using alone/concealing use), increased cleanliness/hygienic use, more transparency between patients and clinicians about drug use, and higher retention in care for inpatients. These results reflect recent research which highlights similar benefits of integrating harm reduction programs into healthcare settings [6, 17, 92, 93]. Harm reduction services, including SIS, may be especially useful in helping to maintain clients in HIV care, and thus provide opportunities for positively impacting the HIV treatment cascade [94, 95].

Our findings also highlight that clients see at least some benefits for those who do not use drugs, specifically around containment of drug use in one area of the hospital, yet concerns also arise, regarding potential triggers for clients who are abstaining from drug use and around hospitals as spaces for recovery. Cortina et al. (2018) also found that PWUD who were trying to abstain from drugs may view hospitals as “protected environments removed from their routine triggers that reinforce drug use” (p.7) [10]. Similar to other feasibility studies, especially those focused on non-PWUD stakeholders [43, 46, 57, 96], issues of morality surface in some of our participants’ discussions regarding their opposition to SIS on the basis that it endorses/supports illicit drug use. Naturally, issues of morality are heightened when healthcare environments, like hospitals, are considered as these institutions wish to avoid being seen as “promoting” behaviour (i.e. illicit drug use) which runs “contrary” to the health of clients. Addressing the needs of patients with complex health problems, including substance use is challenging and not unique to our study setting alone. For example, we describe concerns about how to implement SIS without disrupting care for hospital clients who do not use drugs. This issue is likely to have relevance in settings in the UK and US where integrated HIV care is delivered by multidisciplinary teams to patient populations that include PWUD [97–101]. Stakeholder opinions about integration of harm reduction services such as SIS are likely to vary as we have documented and solutions based on the principle of equity may help ease decision making.

In the literature, rates of willingness to use SIS range from as low as 36% all the way to 100% [38, 39]; however, most studies find that willingness to use potential services is over 60% [33, 37, 42, 48, 63, 102–105].Vancouver-based studies specifically consider willingness to use SIS in hospital settings, with one study finding that 59.4% of participants who smoked crack cocaine would use an in-hospital supervised inhalation room [10], while another study found that 68.2% of participants were willing to access an in-hospital SIS [12]. Our study found a slightly lower rate of PLHIV (*n* = 7; 46.7%) who would be willing to use hospital-based SIS if they were available, with an additional 26.7% (*n* = 4) of participants who were undecided. Other research findings show that reasons to use SIS are markedly similar to those reported by our participants – including protection from overdose; safety from violence, crime, arrest; being able to use indoors; cleanliness/access to clean equipment; and building trusting relationships with professionals and peers [17, 18, 40, 72]. Remarkably, in our study all survey clients who were current injectors (*n* = 11) cited trust in staff as a top reason for their potential willingness to use onsite-SIS, and further focus group and interview discussions revealed that clients believed SIS would deepen this trust by allowing more honest communication about drugs use with health care providers. McNeil et al. (2014), in a study of SIS at a day program for PLHIV, found that implementation of these services created an open and trusting environment thereby improving care for PLHIV clients [17]. The most common reasons found in the literature for not using a potential SIS, are also similar to those cited by our participants, namely having a preferred place to use, lack of privacy and/or confidentiality, fear of arrest, and attempting abstinence [10, 38–40, 42, 103].

While many studies have looked at people who inject drugs and their willingness to use SIS, little is known about the acceptability, facilitators, and barriers for SIS among PLHIV. A few feasibility studies have included PLHIV participants [10, 12, 106] and there has been some post-implementation research conducted at the Dr. Peter Center [17, 72, 73, 107, 108]. Research at the Dr. Peter Center found that SIS and other integrated harm reduction services not only improved HIV treatment outcomes and increased access to antiretroviral therapy, they also alleviated the impact of homelessness and food insecurity [17, 72]. Interestingly, findings from our study show that for PLHIV issues of who should have access to in-hospital SIS was a key point of contention and this discussion seemed to centre on prevention of HIV transmission. For some, access to SIS needed to be restricted to PLHIV since any association with non-PLHIV was risky. Conversely, others claimed the exact opposite, stating that the hospital had an opportunity to expand its mandate and prevent HIV infection by allowing all persons who inject drugs in the community access to SIS.

Our research is unique in that we included both PWUD and non-PWUD hospital clients in all parts of the study. Previous research studies of SIS in hospital settings tend to focus solely on PWUD and/or staff perspectives [10, 12, 72]. Indeed, SIS feasibility studies that include PWUD tend to focus on those who frequent particular harm reduction services (e.g., needle and syringe programs) or are receiving substance use treatment (e.g., methadone) [48, 73]. Some feasibility studies have tried to expand on their inclusion criteria by also talking to those who are at risk of initiating, often injection, drug use [33] or those who injected/used drugs in the past [34]. Acknowledging PWUD perspectives in SIS feasibility research is crucial to ensure that the development of harm reduction services reflects current needs and encourages empowerment among these key stakeholders [53]. For instance, McNeil et al. (2016) in their study of recently discharged against medical advice PWUD show how hospital-based harm reduction programs can promote patient-centered care for this target population [19]. Research on non-PWUD stakeholders, for example community residents, business owners, police offices etc., shows that acceptability for SIS is predicated by a variety of considerations [27, 52, 109], making it crucial to consider how hospital clients who do not use drugs make decisions regarding SIS acceptability. By looking beyond the primary group of PWUD, in this study we were able to capture a variety of opinions regarding how the potential implementation of SIS may affect current service users at the hospital, including those who currently did not and/or had never used drugs. For instance, issues of courtesy stigma and threats of disruptive behaviours, ranging from shouting to violence or sexual harassment from SIS users, are important to address for clients. Other challenges of integrating SIS into a healthcare setting included issues around organizational priorities (i.e., diversion of limited funding, overburdened staff, and potential loss of donor dollars).

The opinions of PWUD regarding SIS have been typically investigated using either large quantitative surveys [33, 46, 48, 53, 61, 62, 102, 104–106] or to a lesser extent using qualitative interviews or focus groups [36, 43, 47, 110]. Fewer SIS feasibility studies take a mixed-methods approach, with many of these encompassing grey literature/research reports [35, 41, 103, 111–113]. However, given the complexity of issues (e.g., legal, economic, cultural) that have arisen in the research of SIS feasibility, investigators particularly emphasize the value of a mixed-methods approach to fully explore these topics [15]. Neither quantitative nor qualitative methods alone were sufficient for answering our research questions. When used in combination, qualitative and quantitative methods complement one another and allow for a fuller analysis, taking advantage of the strengths of each methodology and helping to offsets the limitations of either one used in isolation.

By utilizing a mixed-methods design, we were able to capture a breadth of opinions about SIS feasibility as well as consider the in-depth reasoning and concerns that arose for participants. Our quantitative data allowed us to gain an understanding of the overall acceptability and demand for SIS, it also allowed us to focus our qualitative data collection procedures and refine the questions we asked in the focus groups and interviews (see Fig. 1). Often times our qualitative data provided nuance and dimension to opinions reported by a large percentage of survey participants, including helping us makes sense of varying levels of support, recognizing key considerations that weighed on clients’ decisions, and providing potential reasoning for undecided responses. While our survey results describe clients’ levels of acceptability and perceptions of demand for SIS, focus group and interview findings expand on this by providing context for why clients are supportive/opposed/undecided about SIS at Casey House across key themes of safety, access, and organizational priorities. Qualitative findings around safety helped to provide further insight into the higher level of acceptability of the inpatient SIS model (e.g., potential to maintain inpatients in care) and why some clients may come to the hospital more often and others less often if SIS is implemented (e.g., SIS provides health benefits for those who use drugs, SIS may be triggering for those in recovery and may create courtesy stigma for those who do not use drugs). The theme of access expanded upon the survey findings related to greater acceptability of an inpatient SIS versus day health program SIS (e.g., inpatients are restricted to the hospital thus in need of hospital-based SIS whereas DHP clients can access SIS elsewhere in the community) and concerns that that demand in the wider community for SIS may overwhelm the hospital. Finally, the theme of organizational priorities provided nuance to survey findings showing high level of perceived demand for hospital-based SIS. Specifically, some participants saw the hospital’s role in addressing the evident needs of clients by providing SIS which may encourage/enable more frequent attendance for some, while others worried about how hospital-based SIS may pull focus away from PLHIV who do not use drugs and may discourage attendance. Moreover, beyond seeing general levels of SIS acceptability, we learned that an integrated approach that links clients to other essential social, health, harm reduction, and addiction services [36, 72] was highly valued. Other times quantitative data allowed us to gain perspective on some strongly held opinions around how drastically or not attendance at the hospital may change if SIS is implemented.

### Limitations

Several limitations of our study warrant consideration. First, due to our non-random sampling method, the results cannot be generalized to the general population of Casey House clients. However, given that our survey reached just under half of all current clients we are confident in assessing a broad sample of opinions. Moreover, our qualitative findings provide a data-rich and nuanced profile of potential SIS users and other hospital clients (although based on a small sample size, especially with regards to gender diversity), with varying opinions on SIS. Second, we relied on self-report data which may be subject to recall and social desirability bias. Nevertheless we sought to overcome this by building rapport with participants and creating a comfortable environment where, as our findings show, most participants were outspoken about highly stigmatizing opinion/experiences [114]. Previous findings also support the validity of self-report data from PWUD [115, 116]. Finally, we recognize that research about hypothetical services may not actually indicate future behaviour [62], yet research has shown that PWUD’s reported willingness to use potential SIS reflect levels of uptake of services post-implementation [117].

## Conclusions

This mixed-methods study examines the feasibility and acceptability of SIS in a hospital setting that caters to PLHIV as well as the concerns and challenges that may arise for non-PWUD who use services in this environment. Our findings lend support to previous research on SIS feasibility, and extend these ideas to a unique population and setting. We draw attention to key issues of safety, access, and organizational priorities that are important to consider for implementing harm reduction strategies, such as SIS, in healthcare settings. The findings from this study helped inform the organization and their decision to apply to the Federal government for the necessary exemption to SIS for clients at the hospital. Given the increased risks of morbidity and mortality for PLHIV who inject drugs [21–23] as well as the problems in retaining them in care in a hospital setting, SIS is a key component of improving care for this marginalized group. Our data showed widespread support of, and need for, hospital-based SIS among client stakeholders; however, attempts to reduce negative impacts on non-drug using clients need to be considered in the balance of implementation plans. Future research should consider harm reduction programs for other modes of drug consumption – especially smoking – that may raise different issues in hospital settings, as well as issues of drug diversion and safer supply, including legal and ethical concerns, which will inevitably come up with the expansion of harm reduction services in hospital settings.

## Supplementary Information

**Additional file 1.** Client Survey. This is the survey developed for and used in this study.

**Additional file 2.** Focus Group Guide. This is the focus group guide developed for this study and used to guide discussion during the client focus groups.

**Additional file 3.** Interview Guide. This is the interview guide developed for this study and used to guide discussion during the client interviews.

## Data Availability

The datasets generated and/or analyzed during the current study are not publicly available because we conducted research with a marginalized social group who are typically distrustful of researchers. As a condition of consent, we agreed not to share data with anyone outside of those named as part of the research team. This approach is typical in research with and for people who use drugs. Further it is challenging to anonymize qualitative data in the contexts are described which can be identifying in nuanced ways.

## Abbreviations

PWUD: People who use drugs
HIV: Human immunodeficiency virus
SIS: Supervised injection services
PLHIV: People living with HIV/AIDS
NIMBY: Not in my backyard
DHP: Day health program
FG#: Focus group number
Int#: Interview number
UK: United Kingdom
US: United States
