# Supplementary material for: Feasibility, acceptability, concerns, and challenges of implementing supervised injection services at a specialty HIV hospital in Toronto, Canada: perspectives of people living with HIV
Source: BMC Public Health. 2021 Jul 29;21:1482. doi: 10.1186/s12889-021-11507-z (PMC8323264; doi:10.1186/s12889-021-11507-z)
Supplement: Supplementary file 1 — Additional file 1. Client Survey. This is the survey developed for and used in this study. [file 12889_2021_11507_MOESM1_ESM.docx]

**Client Survey: Research Study on the Potential Need for Supervised Injection Services (SIS) at Casey House**

Q1 Please note that supervised injection services may be shortened to "SIS".  

We want to survey a broad range of stakeholders in the Casey House community about supervised injection services. In this survey we are reaching out to clients at Casey House. Staff, volunteers, managers and board members are answering similar surveys, with slightly different questions. **Please check which of the following currently best describes you:**

- Client in the day health program
- Client in the inpatient program
- Other, please specify: ________________________________________________

Q2 Here we will ask you about your **gender identity**. Please note that "cis" gender refers to when your gender identity matches the sex you were assigned at birth. Do you identify as:

- Cis male
- Cis female
- Transgender (M to F)
- Transgender (F to M)
- Other, please specify: ________________________________________________
- Prefer not to answer the question

Q3 How many years have you been a client at Casey House?

- Number of years: ________________________________________________
- Prefer not to answer the question

Q4 Next we will ask about your knowledge and opinions about SIS.
   How much do you know about supervised injection services? (Check the closest answer)

- **Very little or no knowledge** (I haven't heard the term before or I know the term but not a lot about what is involved)
- **Some knowledge** (I know something about services or programming offered by SIS)
- **Average knowledge** (I know something about programming offered and about research evidence about the effectiveness of SIS)
- **Fairly knowledgeable** (I know details about programming offered and evidence about the effectiveness of SIS, and I have followed the news about setting up new SIS)
- **Very knowledgeable** (I know details about programming offered and evidence supporting SIS, I have followed the news about setting up new SIS, and I have either done some extra reading or know people who work at or have used SIS)
- Prefer not to answer the question

Supervised injection services are health services that provide a hygienic space for people to come and inject their pre-obtained drugs under the supervision of medically trained staff. In addition to supervised injection, individuals are provided with sterile injection supplies (needles, syringes, cotton, cooker, water, etc.), education on safer injection, overdose prevention and intervention, as well as referrals to various health and social services (e.g., counselling services, drug treatment, housing, income support). At Casey House, supervised injection services in the Day Health Program would potentially be set up in a separate space on the 1st or 2nd floor. Supervised injection services for inpatients could either be in a separate space on the 3rd floor or at the client’s bedside.

Q5 Whatever your current level of knowledge about supervised injection services, we want to know what your thoughts are about SIS in the questions that follow. First we will start with a few statements about supervised injection services and ask you if you agree or not.

I think that supervised injection services should be made available as part of **overall healthcare in Ontario** for individuals who inject drugs:

- Agree
- Disagree
- Haven't made up my mind
- Prefer not to answer the question

Q6 I think that supervised injection services should be made available **at Casey House** **for clients in the day health program:**

- Agree
- Disagree
- Haven't made up my mind
- Prefer not to answer the question

Q7 I think that supervised injection services should be provided **at Casey House for clients in the inpatient program**:

- Agree
- Disagree
- Haven't made up my mind
- Prefer not to answer the question

Q8 The following questions ask about non-medical use of substances. These include substances not prescribed to you by a physician (or other health care provider) and/or substances which you use in ways other than how they were prescribed.  
Have you **ever injected drugs** for non-medical reasons?

- Yes
- No
- Prefer not to answer the question

Q9 Have you injected drugs for non-medical reasons **in the past 6 months**?

- Yes
- No
- Prefer not to answer the question

Q10 In the **past 6 months**, mark which of the following you have experienced: (Please check all that apply)

- I have injected drugs for pleasure
- I have used a needle that had been used before by someone else
- I have used other injection equipment (e.g., cooker, water, filter) that had been used before by someone else
- I have injected drugs to help manage my physical pain
- I have injected drugs to help manage my emotional or psychological problems
- I have had an abscess/infection as a result of my injection
- I have had a collapsed vein as a result of my injection
- I have experienced an opioid overdosed
- None of the above

Q11 In the past 6 months, mark which of the following you have experienced: (Please check all that apply)

- I have witnessed an overdose
- I have injected outside or in a public place (e.g., stairwell, parking garage, public washroom)
- I have injected at Casey House
- I was robbed or attacked while injecting
- I was rushed when trying to prepare a hit
- I was hassled by the police because of my drug use
- I had naloxone when it was needed
- I have used SIS (e.g., The Works, Fred Victor, South Riverdale, Parkdale/Queen West, Moss Park, Regent Park, Street Health, St. Stephen's)
- None of the above

Q12 Have you injected drugs for non-medical reasons **in the past 30 days**?

- Yes
- No
- Prefer not to answer the question

Q13 What **type of drugs** have you injected in the **past 30 days** (check all that apply):

- Opioids (e.g., heroin, methadone, percocet/percodan, dilaudid, fentanyl, opium, morphine, codeine, demerol, oxys, etc.)
- Stimulants (e.g., cocaine, crack, crystal meth, speed, uppers, Ritalin, etc.)
- Other, please specify: ________________________________________________
- Prefer not to answer the question

Q14 I would **use** supervised injection services at Casey House:

- Agree
- Disagree
- Haven't made up my mind
- Prefer not to answer the question

Q15 For what reasons would you potentially **use** SIS at Casey House? (Please check all that apply)

- I would be able to get clean sterile injection equipment
- Overdoses can be prevented or treated
- I can have my drugs tested before use
- I would be able to inject indoors and not in a public space
- I would be safe from crime
- I would be safe from being seen by the police
- I would be able to get referrals for other services
- Casey House is a place I trust/ the staff at Casey House know me
- Other, please specify: ________________________________________________
- Prefer not to answer the question

Q16 For what reasons would you potentially **not use** SIS at Casey House? (Please check all that apply)

- I already have a preferred place to inject
- I do not want people to know I use drugs
- I'm afraid my name will not remain confidential and this will become part of my health record
- SIS at Casey House is too far away for me to travel
- I fear being caught with drugs by the police
- I do not trust supervised injection services
- I can get clean sterile needles and other supplies already
- I feel there are too many rules at supervised injection services
- I currently do not inject drugs
- Other, please specify: ________________________________________________
- Prefer not to answer the question

Q17 The next set of questions will ask about your knowledge of Casey House clients’ non-medical use of substances. I have **heard** of Casey House clients **injecting drugs for non-medical reasons** (check all that apply):

- In their rooms (inpatient clients)
- Somewhere else within Casey House (e.g., bathrooms, stairwells, etc.)
- Close to Casey House (e.g., outside, etc.)
- No, I have not
- Prefer not to answer the question

Q18 How often do you **hear** about Casey House clients **injecting drugs for non-medical reasons** in or near the building?

- Daily (3 to 7 times per week)
- Weekly (1 to 2 times per week)
- Monthly (less than 3 times per month)
- Less often
- Prefer not to answer the question

Q19 I have **seen** Casey House clients **injecting drugs** **for non-medical reasons** (check all that apply):

- In their rooms (inpatient clients)
- Somewhere else within Casey House (e.g., bathrooms, stairwells, etc.)
- Close to Casey House (e.g., outside, etc.,)
- No, I have not
- Prefer not to answer the question

Q20 How often have you **seen** Casey House clients **injecting drugs for non-medical reasons** in or near the building?

- Daily (3 to 7 times per week)
- Weekly (1 to 2 times per week)
- Monthly (less than 3 times per month)
- Less often
- Prefer not to answer the question

Q21 I would **recommend** supervised injection services at Casey House to a **friend** who is also a client and who injects drugs:

- Agree
- Disagree
- Haven't made up my mind
- Prefer not to answer the question

Q22 How would the existence of supervised injection services at Casey House **impact how often you come** to Casey House?

- I would come more often
- I would come less often
- It would not impact how often I come
- Prefer not to answer the question
