# Supplementary material for: Feasibility, acceptability, concerns, and challenges of implementing supervised injection services at a specialty HIV hospital in Toronto, Canada: perspectives of people living with HIV
Source: BMC Public Health. 2021 Jul 29;21:1482. doi: 10.1186/s12889-021-11507-z (PMC8323264; doi:10.1186/s12889-021-11507-z)
Supplement: Supplementary file 2 — Additional file 2. Focus Group Guide. This is the focus group guide developed for this study and used to guide discussion during the client focus groups. [file 12889_2021_11507_MOESM2_ESM.docx]

**Focus Group Discussion Guide – Client**

**2019**

*Now that we have heard and talked about the operation of SIS, we would like to talk more about your thoughts regarding the potential need for SIS at Casey House, as part of the DHP.*

1. What role, if any, do you think Casey House has to play in addressing drug-related harms within its community?

- Do you think this role includes providing SIS as part of the DHP?

1. What were your first impressions of the SIS demo room?
2. What do you think might be the potential benefits of SIS for…

- DHP clients who inject drugs?
- DHP clients who DON’T inject drugs?
- Casey House inpatients?
- Staff?
- Surrounding neighbours/community?

1. What do you think might be the consequences/drawbacks of SIS for …

- DHP clients who inject drugs?
- DHP clients who DON’T inject drugs?
- Casey House inpatients?
- Staff?
- Surrounding neighbours/community?

1. What barriers, if any, do foresee to providing SIS in the …

- DHP?

1. Now I’m going to focus on a SIS within the DHP
   1. Do you think a SIS will be used by DHP clients who inject drugs? Why/why not?
   2. Will a SIS encourage these clients to use Casey House more often?
   3. How could the program be designed to encourage use of the SIS by DHP clients?
   - Location; entrance; privacy;
   - Chill out space
   - Hours available; women only hours
   - Confidentiality
   - Rules within the space, including assisted injection
   - Staffing model

7) What impact, if any, do you think a SIS might have on how clients who DON’T inject drugs use Casey House?

- If yes/no, please explain?

8) What kinds of things should be measured to determine if SIS at Casey House is operating successfully?

- As part of the DHP?
- As part of the inpatient program?

9) How did you feel about the process that we used for this study (so we had a walkthrough the demo SIS room, had presentations on evidence, had staff member from an existing SIS present their experiences, allowed for Q&A, and then had a group discussion)?

- Did you like this format?
- What worked/what didn’t work?
- Did the extra information help you form your opinions about SIS?

10) Did you feel that the research team tried to sway your opinion at all during the study? How so?
