# Supplementary material for: Feasibility, acceptability, concerns, and challenges of implementing supervised injection services at a specialty HIV hospital in Toronto, Canada: perspectives of people living with HIV
Source: BMC Public Health. 2021 Jul 29;21:1482. doi: 10.1186/s12889-021-11507-z (PMC8323264; doi:10.1186/s12889-021-11507-z)
Supplement: Supplementary file 3 — Additional file 3. Interview Guide. This is the interview guide developed for this study and used to guide discussion during the client interviews. [file 12889_2021_11507_MOESM3_ESM.docx]

**Interview Discussion Guide – Client**

**2019**

*Now that we have talked about the operation of SIS, we would like to talk more about your thoughts regarding the potential need for SIS at Casey House, as part of the inpatient program.*

1. What role, if any, do you think Casey House has to play in addressing drug-related harms within its community?

- Do you think this role includes providing SIS as part of the inpatient program?
- Does this include providing bedside injection?

1. What were your first impressions of the SIS demo room pictures?
2. What do you think might be the potential benefits of SIS for…

- Inpatient clients who inject drugs?
- Inpatient clients who DON’T inject drugs?
- DHP clients?
- Staff?
- Surrounding neighbours/community?

1. What do you think might be the consequences/drawbacks of SIS for …

- Inpatient clients who inject drugs?
- Inpatient clients who DON’T inject drugs?
- DHP clients?
- Staff?
- Surrounding neighbours/community?

1. What barriers, if any, do foresee to providing SIS in the …

- Inpatient program?

1. Now I’m going to focus on SIS for the inpatients at Casey House
   1. Do you think a SIS will be used by inpatients who inject drugs? Why/why not?
   2. How could the program be designed to encourage use of the SIS by inpatient clients?
   - Location; bedside
   - Privacy/confidentiality
   - Hours available
   - Rules, including assisted injection
   - Staffing model

7) What impact, if any, do you think a SIS might have on how clients who DON’T inject drugs use Casey House?

- If yes/no, please explain?

8) What kinds of things should be measured to determine if SIS at Casey House is operating successfully?

- As part of the DHP?
- As part of the inpatient program?

9) How did you feel about the process that we used for this study (so we showed you pictures of a demo SIS room, had presentations on evidence, allowed for Q&A, and then had a discussion)?

       Did you like this format?

       What worked/what didn’t work?

       Did the extra information help you form your opinions about SIS?

10) Did you feel that the research team tried to sway your opinion at all during the study? How so?
